# Quality of life and its predictors among breast cancer patients treated with surgery—a retrospective minimum 3-year follow-up study

**DOI:** 10.3389/fonc.2024.1466625

**Published:** 2024-11-25

**Authors:** Wen-Zhen Tang, Yao-Qiong Lu, Sheng-Rui Zhu, Yan-Juan Teng, Tian-Fu Wei, Guo-Lian Chen, Kui Jia

**Affiliations:** ^1^ Department of Hepatobiliary Surgery, The First Affiliated Hospital of Guangxi Medical University, Nanning, China; ^2^ Department of Geriatrics Cardiology, The First Affiliated Hospital of Guangxi Medical University, Nanning, China; ^3^ Department of Gastrointestinal Surgery, The First Affiliated Hospital of Guangxi Medical University, Nanning, China; ^4^ Department of Oncology, The First Affiliated Hospital of Guangxi Medical University, Nanning, China

**Keywords:** breast cancer, follow-up, quality of life, surgery, factor

## Abstract

**Aim:**

Quality of life (QoL) has been identified as an important indicator of positive outcomes among breast cancer (BC) survivors. However, the status and predictors of QoL in China remain unclear. This retrospective follow-up study aimed to examine the QoL levels among BC patients following surgery and to assess the influence of sociodemographic, clinical, and psychological factors on QoL.

**Methods:**

An institution-based retrospective follow-up study was conducted among 714 BC patients who received surgery at the First Affiliated Hospital of Guangxi Medical University between January 2016 and December 2019. Our primary outcome measure was QoL, assessed using the European Organization for Research and Treatment of Cancer QoL Questionnaire Core 30 (EORTC QLQ-C30). Anxiety and depression were evaluated by the Self-Rating Anxiety Scale (SAS) and the Self-Rating Depression Scale (SDS), respectively. Data on the patient demographics and clinical were derived from medical records. Results are presented as means (SD), medians [Q1, Q3], or percentage (%). We used R 4.2.2 software to identify factors associated with QoL after BC surgery. AMOS 28.0 was used to construct a structural equation model (SEM) to predict QoL outcomes.

**Results:**

The overall QoL score was 43.30 ± 4.77 (mean ± SD). Predictive factors were: surgery type, radiotherapy, anxiety, and depression (*p*<0.05). The results of the SEM indicated that anxiety and depression had a direct negative effect on QoL (effect value was -0.46, -0.84, respectively, *p*<0.05), radiotherapy had a direct positive effect on QoL (effect value was 0.71, *p*<0.05). The type of surgery (mastectomy) impacted QoL both directly and indirectly through its association with depression, with direct and indirect effect values of -0.96 and -0.66, respectively (*p* < 0.05).

**Conclusion:**

The QoL of BC patients after surgery is generally moderate to low. Medical staff should prioritize early identification and rehabilitation management for patients experiencing anxiety, depression, radiotherapy, and mastectomy to enhance their QoL. Our findings provide a strong foundation for developing nursing intervention plans and assessment guidelines for practitioners caring for BC patients.

## 1 Introduction

Breast cancer (BC) is the most common cancer in women worldwide (1). The Global Cancer Statistics Report 2022 indicates there were 2.3 million new cases of BC, representing 11.6% of all cancer cases, along with1.67 million of total deaths (2). In 2022, the number of new cases of BC in China was 357,000, accounting for 23.81% of female cancer cases; the number of BC deaths in China was 75,000, making up 15.4% of female cancer deaths (3).

Currently, surgery is the primary treatment for BC (4). As a result of breast surgery, BC patients often experience significant changes in appearance, including breast deformities and surgical scars. Breasts are considered a symbol of femininity and an important part of a woman’s identity. Consequently, any resulting alterations after surgery can adversely affect body image perception (5), leading to a decline in quality of life (QoL) (6). A recent study by Rosenberg found that BC patients may experience anxiety, depression, stigma, and other negative emotions due to changes in body image and femininity, which can furthermore diminish their QoL (7).

With the advancements in modern medical treatment, the survival time of BC patients is also increasing. Reports from European and American countries indicate that the relative survival rates of female BC patients are 95% for one year, 80.4% for five years, and 73.4% for ten years. Additionally, the five-year survival rates of early-stage BC patients approach 100% (8). In China, the five-year survival rate for women with BC is 73% (9). In conclusion, the prognosis for BC is relatively favorable.

As the survival rates of BC patients continue to improve, medical staff should focus not only on prolonging life but also on enhancing the QoL and meeting patients’ overall prognostic needs (10). Therefore, the QoL of patients with BC after surgery has garnered increasing attention. QoL is defined as how individuals from various cultures and value systems perceive their well-being in relation to their goals, expectations, standards, and concerns (11). QoL is a multidimensional construct that encompasses physiological, psychological, social, and behavioral aspects, and the overall health status (12). The QoL of BC patients is affected by various factors, including sociodemographic, clinical, psychological and other factors (13–15). Predictors of QoL can vary significantly from one study to another.

Previous studies on the QoL of postoperative BC patients mainly focused on hospitalized patients, with a notable lack of follow-up studies involving discharged patients (16, 17). In addition, the relevant studies were mainly conducted in Western countries and may not be generalized to the Chinese population due to cultural and clinical differences. Identifying the predictors that contribute to the poor QoL of Chinese patients after BC surgery will enable targeted interventions to improve their QoL.

The first objective of this study was to examine the level of QoL in BC patients after surgery in China. Our second aim is to identify sociodemographic, disease, and psychological factors that influence the QoL and construct its predictive models using a structural equation model (SEM).

## 2 Materials and methods

### 2.1 Study design and area

An institution-based retrospective follow-up study was conducted to identify the predictors of QoL after surgery in BC patients at the First Affiliated Hospital of Guangxi Medical University, China. Medical data were collected from July 2022 to September 2022. The First Affiliated Hospital of Guangxi Medical University is a general hospital with over 2,700 beds, featuring 46 clinical departments, 71 wards, and 19 medical technical departments. In 2022, the hospital recorded approximately 3.88 million outpatient emergency visits, 135,400 discharges, and 72,900 surgical procedures, with an average length of stay of 7.26 days. The hospital provides services in various fields, including psychiatry, surgery, laboratory services, and pharmacy. Our study specifically took place in the Department of Gastrointestinal and Gland Surgery, which has 50 beds and is recognized as a key clinical specialty in China, a priority clinical specialty project in Guangxi, and a significant medical and health discipline in the region.

### 2.2 Population

All BC patients admitted to the First Affiliated Hospital of Guangxi Medical University for surgery from January 2016 to December 2019 were included in our study.

#### 2.2.1 Inclusion criteria

All adult BC patients who received surgery at the First Affiliated Hospital of Guangxi Medical University between 2016 and 2019 were included in the study.

#### 2.2.2 Exclusion criteria

Patients with existing tumors or incomplete clinical data were excluded.

### 2.3 Research instruments

#### 2.3.1 Demographic characteristics

The demographic characteristics of the subjects, including sex, age, education, marital status, medical insurance status, residence, and employment status.

#### 2.3.2 Clinical characteristics

The clinical characteristics of the subjects were examined, including variables such as history of smoking and alcohol consumption, history of hypertension and diabetes, type of surgery, duration of the operation, clinical stage, length of stay, and whether patients received chemotherapy, radiotherapy, or neoadjuvant chemotherapy. The clinical features collected in this study are detailed in **Supplementary Table S1**.

#### 2.3.3 Quality of life

Quality of life was measured as the European Organization for Research and Treatment of Cancer QoL Questionnaire Core 30 (EORTC QLQ-C30), developed by the European Organization for Research and Treatment for Cancer (EORTC) (18). It consists of 30 items measuring a global health scale (GHS), five functional scales (physical, role, emotional, cognitive, and social functioning), and nine symptom scales (fatigue, nausea and vomiting, pain, dyspnea, insomnia, loss of appetite, constipation, financial difficulties and diarrhea). Each dimension generates a score and the score ranges from 0 to 100, with a higher value representing a higher level of QoL (18).

#### 2.3.4 Anxiety

Anxiety was evaluated by the Self-Rating Anxiety Scale (SAS), developed by Zung in 1971 (19). The total SAS score ranges from 20 to 80 points. The higher the SAS score, the higher level of the anxiety. The severity of anxiety can be divided into the following four categories: no anxiety (score<50), mild anxiety (score 50-59), moderate anxiety (score 60-68), and severe anxiety (score≧69) (20).

#### 2.3.5 Depression

The depression was assessed by using the Self-Rating Depression Scale (SDS), compiled by Zung (21). The SDS scale consists of 20 items, each item corresponds to a symptom of interest and is scored on a 1-4 scale. The final score is calculated by dividing the cumulative score for each item by 80. A score of less than 0.5 indicates no depression, while a score greater than or equal to 0.5 is considered depression.

### 2.4 Data collection

The data and were collected from July 2022 to September 2022. The demographic and clinic variable were extracted from the patient record, medical history sheets, and surgical notes in the hospital system. We collected anxiety, depression, and QoL scores through telephone follow-up interviews. The patient list was reviewed in advance, and participants who agreed to participate in the study were recruited after verbally describing the purpose and procedure of the study to the subjects over the phone. The researchers then conducted the survey, which took about 15 to 25 minutes to complete.

The data collection process was supervised by two head nurses with extensive clinical experience and psychological qualifications. Before data collection, data collectors were given a half-day training on how to collect data from patient records and how to ask patients for information about the scale. During the data collection process, the researcher checks the data integrity in time. Finally, the data is cleaned and cross-checked before analysis.

### 2.5 Data analyses

After data collection was completed, the data was entered and error cleared using Excel, and then the data was exported to R 4.1.1 and AMOS 24.0 for analysis. Descriptive statistics were performed using numbers and percentages (%) for categorical variables, and continuous variables are expressed as mean ± standard deviation (SD), and median (interquartile distance). The variables included in the model were preliminarily determined by one-way analysis of variance, Pearson correlation analysis, and multivariate analysis. AMOS 24.0 was used to construct the structural equation model (SEM), and the maximum likelihood method was used to estimate the parameters. The following indices were recommended to evaluate model fit: Root Mean Square Error of Approximation (RMSEA), goodness of fit index (GFI), Tucker-Lewis index (TLI), normed fit index (NFI), and comparative fit index (CFI) greater than 0.90 and the Chi-square freedom ratio (CMIN/DF) of 3 or less. *P*<0.05 or P< 0.01 indicates a statistically significant difference.

## 3 Results

### 3.1 Quality of life in patients with breast cancer after surgery

In our study, the QoL score of BC patients was moderate to low level, and the mean ± SD score of the overall QoL was 43.30 ± 4.77. **Table 1** describes the mean scores, median and percentile range of total score, physical function, role function, emotional function, cognitive function, social function, global health status, financial difficulty and symptom domain. The dimension that scored highest was social function (98.80 ± 3.36), followed by role functioning (97.88 ± 5.93) and cognitive functioning (97.63 ± 7.97).

**Table 1:** The overall QoL and domain-specific QoL scores of the study population.

Dimension	Mean score (SD)	25th percentile	75th percentile
Total score	43.30 (4.77)	41	44.5
Physical function	86.98 (63.15)	93.33	93.33
Role function	97.88 (5.93)	100.00	100.00
Emotional function	97.60 (5.25)	100.00	100.00
Cognitive function	97.63 (7.97)	100.00	100.00
Social function	98.80 (3.36)	100.00	100.00
Global health status	35.04 (5.45)	35.71	35.71
Financial difficulty	95.30 (17.04)	100.00	100.00
Symptom domain	8.52 (9.52)	4.17	8.33

### 3.2 Univariate analysis of QoL after breast cancer surgery

The average score of the QoL in this study is 43 points. Therefore, we used this value as a cut-off point, and 714 BC patients can be divided into two groups: high level and low level of QoL. The overall QoL scores were considered dependent variables and sociodemographic, clinical, and psychological factors collected were considered independent variables. The results of univariate analysis showed that patients with high levels of QoL had a statistically significant difference in length of stay (*P*<0.001), surgery type (*P*<0.001), radiotherapy (*P*=0.06), operation time (*P*=0.007), depression (*P*<0.001) and anxiety (*P*<0.001), compared with patients with low level of QoL (*P*<0.05) (**Table 2**).

**Table 2:** Comparison of different QoL scores in 714 breast cancer patients.

Variables	Total (n = 714)	Low level of QoL (n = 498)	High level of QoL (n = 216)	*p-*value
Age, Median (Q1, Q3)	51 (44.25, 58)	51 (44, 58)	52 (45.75, 59)	0.308
Education, N (%)				0.891
Middle School and Below	443 (62)	308 (62)	135 (62)	
High School	89 (12)	64 (13)	25 (12)	
Bachelor Degree or Higher	182 (25)	126 (25)	56 (26)	
Marital, N (%)				0.202
Married	645 (90)	455 (91)	190 (88)	
Single/Divorced/Widowed	69 (10)	43 (9)	26 (12)	
Residence, N (%)				0.334
Urban	402 (56)	274 (55)	128 (59)	
Rural	312 (44)	224 (45)	88 (41)	
Full employment, N (%)				0.455
No	197 (28)	142 (29)	55 (25)	
Yes	517 (72)	356 (71)	161 (75)	
Having insurance, N (%)				0.426
No	24 (3)	19 (4)	5 (2)	
Yes	690 (97)	479 (96)	211 (98)	
Length of stay, Median (Q1, Q3)	13 (10, 16)	12 (10, 15)	13 (11, 17)	<0.001
History of smoking, N (%)				0.558
No	711 (100)	495 (99)	216 (100)	
Yes	3 (0)	3 (1)	0 (0)	
History of Drinking, N (%)				0.674
No	708 (99)	493 (99)	215 (100)	
Yes	6 (1)	5 (1)	1 (0)	
Neoadjuvant chemotherapy, N (%)				0.199
No	633 (89)	447 (90)	186 (86)	
Yes	81 (11)	51 (10)	30 (14)	
Hypertension, N (%)				0.416
No	646 (90)	454 (91)	192 (89)	
Yes	68 (10)	44 (9)	24 (11)	
Diabetes, N (%)				0.642
No	682 (96)	474 (95)	208 (96)	
Yes	32 (4)	24 (5)	8 (4)	
Surgery Type, N (%)				<0.001
Breast Reconstruction	125 (18)	72 (14)	53 (25)	
Mastectomy	565 (79)	420 (84)	145 (67)	
Breast Conservation	23 (3)	6 (1)	17 (8)	
Operation time, Median (Q1, Q3)	145 (114, 195)	141 (112.25, 185)	149 (118.5, 217)	0.007
Clinical-Stage, N (%)				0.666
I	199 (28)	143 (29)	56 (26)	
II	349 (49)	245 (49)	104 (48)	
III	162 (23)	107 (21)	55 (25)	
IV	4 (1)	3 (1)	1 (0)	
Chemotherapy, N (%)				0.713
No	96 (13)	69 (14)	27 (12)	
Yes	618 (87)	429 (86)	189 (88)	
Radiotherapy, N (%)				0.006
No	423 (59)	312 (63)	111 (51)	
Yes	291 (41)	186 (37)	105 (49)	
Depression, Median (Q1, Q3)	43 (42, 43)	43 (42, 43)	42 (39, 43)	<0.001
Anxiety, Median (Q1, Q3)	35 (32, 36)	35 (34, 36)	32 (28, 34)	<0.001

### 3.3 Correlation analysis of QoL after breast cancer surgery

The results of correlation analysis showed that radiotherapy (*P*<0.01), anxiety (*P*<0.01), and depression (*P*<0.01) were significantly correlated with QoL after surgery. Higher levels of QoL scores were observed in patients with radiotherapy, while lower level of QoL scores was associated with higher levels of depression and anxiety (**Table 3**).

**Table 3:** Variables of QoL in patients with breast cancer by correlation analysis.

Variables	Correlation value	95%CI	*p* value
Age	0.03	(-0.05, 0.10)	0.49
Education	<0.01	(-0.07, 0.07)	1.00
Marital	0.05	(-0.02, 0.13)	0.16
Residence	-0.04	(-0.11, 0.03)	0.29
Full employment	0.03	(-0.04, 0.10)	0.40
Having insurance	0.04	(-0.04, 0.11)	0.31
History of smoking	-0.04	(-0.12, 0.03)	0.25
History of drinking	-0.03	(-0.10, 0.05)	0.47
Neoadjuvant chemotherapy	0.05	(-0.02, 0.13)	0.16
Hypertension	0.04	(-0.04, 0.11)	0.34
Diabetes	-0.02	(-0.10, 0.05)	0.51
Surgery type	-0.04	(-0.11, 0.04)	0.32
Clinical stage	0.04	(-0.03, 0.11)	0.27
Chemotherapy	0.02	(-0.06, 0.09)	0.63
Radiotherapy	0.11	(0.03, 0.18)	<0.01
Depression	-0.38	(-0.44, -0.31)	<0.01
Anxiety	-0.48	(-0.54, -0.43)	<0.01

### 3.4 Multivariate analysis of QoL after breast cancer surgery

Factors that were statistically significant in the univariate analysis were included in the multifactorial analysis. The results showed that radiotherapy (OR=1.76, 95%CI: 1.10-2.82), surgery type (mastectomy) (OR=0.26, 95%CI: 0.10-0.65), depression (OR=0.62, 95%CI: 0.55-0.71) and anxiety (OR=0.64, 95%CI: 0.58-0.70) were predictors of QoL (**Table 4**). However, length of stay and operation time had no statistical significance with the QoL(*P*>0.05).

**Table 4:** Variables of QoL in patients with breast cancer by multivariate analysis.

Variables	Estimate	Std Error	Z value	P value	OR	95%CI
Length of stay	0.01	0.03	0.48	0.63	1.10	(0.96-1.08)
Operation time	<0.01	<0.01	0.48	0.63	1.00	(0.99-1.00)
Surgery type (mastectomy)	-1.35	0.47	8.19	<0.01	0.26	(0.10-0.65)
Radiotherapy	0.57	0.24	2.37	0.02	1.76	(1.10-2.82)
Depression	-0.47	0.07	-7.14	<0.01	0.62	(0.55-0.71)
Anxiety	-0.45	0.05	-9.78	<0.01	0.64	(0.58-0.70)

### 3.5 Structural equation model for predicting QoL after breast cancer surgery

Based on the results of univariate analysis, correlation analysis, and multivariate analysis, the SEM of predictive of QoL after BC surgery was constructed. The maximum likelihood ratio method was used to fit the data of the research model, and the fitting indicators of the above hypothesis model were poor. After multiple adjustments, the two variables of operation time and length of stay were deleted. After adjustment, all the fitting indexes of the model were good, and the CMIN/DF was 1.447 (ideal value ≤ 3). The RMSEA was 0.026 (ideal value < 0.05), the TLI was 0.970 (ideal value >0.90), the CFI was 0.990 (ideal value >0.90), and the NFI was 0.971 (ideal value >0.90) (**Table 5**).

**Table 5:** Fitting index of the research model.

The goodness of fit indices	Cut-off value	Results	Status
CMIN/DF	≤3	1.477	Acceptable
RMSEA	<0.05	0.026	Acceptable
TLI	>0.90	0.970	Acceptable
CFI	>0.90	0.990	Acceptable
NFI	>0.90	0.971	Acceptable

**Figure 1:**
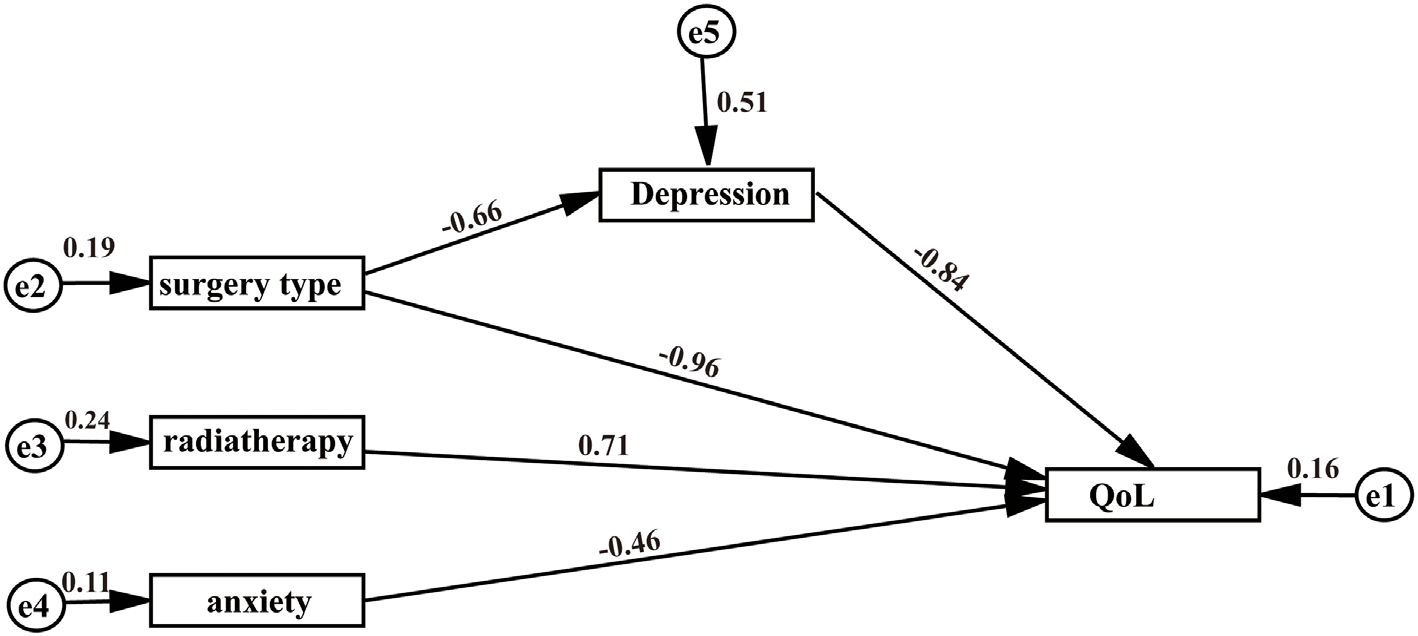
SEM of predictors on QoL of breast cancer patients after surgery.

The results of the model suggest that radiotherapy, anxiety and depression directly affect the QoL of BC patients after surgery. Furthermore, type of surgery can not only directly affect the QoL of BC patients after surgery, but also affect the QoL of BC patients after surgery through the mediating effect of depression, as shown in **Figure 1** and **Table 6**.

**Table 6:** Results of the path coefficients in the model.

Paths	Estimate	Standard error	C.R	P value
Surgery type →Depression	-0.657	0.196	-3.345	<0.01
Depression → QoL	-0.837	0.067	-12.553	<0.01
Radiotherapy → QoL	0.710	0.308	2.307	0.021
Anxiety → QoL	-0.457	0.046	-10.008	<0.01
Surgery type →QoL	-0.961	0.352	-2.728	<0.01

## 4 Discussion

This hospital-based retrospective follow-up study investigated the QoL level and its predictive factors of BC patients after surgery in Guangxi, China, and constructed a SEM of the predictors of QoL of BC patients. Using a questionnaire-based follow-up design and data collected at the patient level using a validated instrument (QLQ-C30), it is reasonable to conclude that this study selected a representative sample of BC patients who had undergone surgical treatment in the Guangxi, China. To our knowledge, this is the first time this study has been conducted in the Guangxi, China. The study focused on the QoL of patients 3-6 years after surgery, aiming to provide a theoretical basis for enhancing the QoL of BC patients. In terms of assessing the QoL, Chinese researchers mostly focus on BC patients during hospitalization. For example, Li et al. conducted a cross-sectional study on BC patients underwent chemotherapy in China (22). In addition, previous studies mostly used univariate and multivariate analysis to analyze the predictors of QoL (23, 24). However, we not only used univariate and multivariate analysis but also used SEM to analyze the predictors by path analysis further. To the best of our knowledge, this is one of the few studies conducted in China that that explore the QoL level of BC patients 3-6 years after surgery and its predictors.

The overall QoL of the BC patients in this study was moderately below average. The highest score was in social function (98.80 ± 3.36), followed by role function (97.88 ± 5.93) and emotional function (97.60 ± 5.25). Our study found that higher QoL scores were reported from patients with radiotherapy, which is consistent with previous studies (25, 26). Zhang et al. investigated the relationship between the end of radiotherapy and the level of QoL in BC patients and found that role functioning scores, pain symptoms, and concerns about the future improved over time with radiotherapy (25). Juan et al. evaluated QoL in elderly BC patients treated with radiotherapy and results showed that QoL scores decreased at the end of treatment and increased after 6 weeks (27). However, people hold different views on the relationship between radiotherapy and QoL, and some scholars believe that radiotherapy will negatively influence QoL (28, 29). Marceila et al. evaluated the effect of radiodermatitis on the QoL of BC patients during the whole period of radiotherapy and found that radiodermatitis can have a negative impact on the QoL by affecting the symptoms, daily activities, leisure, work, study, and other aspects (28). The main reason for this difference is that the time point of observation varies between studies, with some studies looking at QoL during or after radiotherapy. During radiotherapy, patients may have a series of radiotherapy complications, such as dry mouth and fatigue, but in the long run, radiotherapy can reduce cancer recurrence and metastasis, which has a positive significance for the survival of patients. Therefore, addressing radiotherapy will improve the long-term QoL of BC patients.

Furthermore, we found that mastectomy can affect QoL not only directly but also indirectly by influencing the patient’s level of depression. On the one hand, mastectomy has a direct negative impact on the QoL of patients, as it is often associated with significant physical changes, including the loss of breast tissues (30, 31). Additionally, complications following mastectomy can restrict daily activities and delay a patient’s return to work (32, 33). In our study, people treated with mastectomy demonstrate a lower QoL, which is also found in previous studies (34, 35). Rosenberg et al. conducted a retrospective, multicenter study of 826 young survivors and discovered that patients who underwent mastectomy had poorer body image and sexual behavior over 5 years (7). Compared with mastectomy, breast reconstruction and breast conservation surgery better protect the patient’s body image and can have a positive impact on QoL (30). More specifically, breast reconstruction can help women re-establish a positive self-image, feel more comfortable with their bodies, and motivate them to return to normal lives and work (36). Additionally, breast-conserving surgery offers a significant advantage in maintaining patients’ body image compared to mastectomy (37).

On the other hand, mastectomy indirectly affects patients’ QoL through its mediating effect on depression. Breasts are crucial to a woman’s body image, and the loss associated with mastectomy can lead to feelings of diminished femininity, poor body image, and decreased attraction to partners, ultimately contributing to heightened levels of depression (38). This depressed mood, in turn, leads to a further decline in the patient’s QoL. A meta-analysis conducted in 2020 from Taiwan systematically reviewed nine relevant studies and found that the risk of depression after mastectomy was 1.36 (95% CI, 1.11-1.65), which is negatively associated with QoL (39).

We also found that anxiety and depression decrease the QoL of BC patients, which is supported by most other studies (40, 41). Anxiety and depression are common psychological morbidities in patients with BC after surgery (42). Zhang et al. evaluated the QoL of 71 patients with breast cancer-related lymphedema and found that anxiety was one of the important predictors of QoL (43). In the study by Sophie et al., the QoL of BC patients at the time of BC diagnosis, the end of treatment, and 6 months after the end of treatment were collected, and the psychosocial factors and dimensions affecting the QoL of patients with non-metastatic BC were evaluated. The results showed that anxiety and depression had a negative impact on the QoL of patients (44). Anxiety and depression can lead to physical discomfort, such as insomnia, headache, and muscle tension (45, 46). Moreover, anxiety and depression may lead to a decline in personal social ability, which may cause self-isolation and social avoidance, reducing their QoL (47). This indicates that clinical medical staff should provide support services to timely guide patients’ emotions, encourage patients to vent their negative psychology, and give full spiritual support, which will have a positive impact on the QoL.

The strength of this study lies in its status as the first retrospective follow-up investigation of QoL among BC patients in Guangxi, China. Our findings underscore the importance of focusing on the long-term QoL of BC survivors after surgery. Additionally, we utilized previously validated instruments to assess QoL 3-6 years post-surgery and employed SEM to identify predictors of QoL. An important finding is that the type of surgery can directly impact the QoL of BC patients’ post-surgery, as well as influence it indirectly through the mediating role of depression.

However, several limitations should be acknowledged. First, as a retrospective study, our research is subject to inherent biases, including the inability to control for confounding factors and the limitations of the available data. Second, we collected data from only one hospital, which may introduce potential bias and may not accurately represent the broader population of BC patients. Furthermore, our study population may be inherently biased, as we selected only patients who had undergone BC surgery, survived, and were being followed up.

It is important to note that our follow-up period was set at 3-6 years after surgery, and follow-up questionnaires should be administered at multiple time points, both before and after surgery, to obtain a complete picture of the patient’s experience and its changes in QoL. In addition, this study is only a single-center study, and further multi-center studies can be carried out in the future. Furthermore, future prospective studies are needed to better evaluate the effectiveness of treatment for BC patients after multidisciplinary collaboration and the impact of more specific surgery type on the QoL of BC patients.

## 5 Conclusion

Our study offers valuable insights for healthcare professionals and researchers by analyzing the QoL of BC patients 3-6 years after surgery and identifying its predictors. Our findings indicate that patients experience moderate to low levels of QoL, with mastectomy, radiotherapy, anxiety, and depression identified as significant predictors. Additionally, mastectomy indirectly affects the QoL of BC patients through the mediating role of depression. Future studies with larger sample sizes are necessary to further validate these results and develop comprehensive interventions aimed at improving the QoL of BC patients. Moreover, this study underscores the importance of ongoing prospective research to continually assess the QoL of BC patients.

## Data Availability

The original contributions presented in the study are included in the article/**Supplementary Material**. Further inquiries can be directed to the corresponding author.
