# Supplementary material for: Quality of life and its predictors among breast cancer patients treated with surgery—a retrospective minimum 3-year follow-up study
Source: Front Oncol. 2024 Nov 25;14:1466625. doi: 10.3389/fonc.2024.1466625 (PMC11626211; doi:10.3389/fonc.2024.1466625)
Supplement: Supplementary file 1 [file Table1.docx]

Table S1 The clinical information

| Clinical characteristic | Category |  |
| --- | --- | --- |
| Length of stay (d) | |  |
| History of smoking | |  |
|  | | No |
|  | | Yes |
| History of Drinking | |  |
|  | | No |
|  | | Yes |
| Hypertension | |  |
|  | | No |
|  | | Yes |
| Diabetes | |  |
|  | | No |
|  | | Yes |
| Surgery Type | |  |
|  | | Immediate Breast Reconstruction |
|  | | Mastectomy |
|  | | Breast Conservation |
| Operation time (min) | |  |
| Clinical-Stage | |  |
|  | | Ⅰ |
|  | | Ⅱ |
|  | | Ⅲ |
|  | | Ⅳ |
| Chemotherapy | |  |
|  | | No |
|  | | Yes |
| Radiotherapy | |  |
|  | | No |
|  | | Yes |
| Neoadjuvant Chemotherapy | |  |
|  | |  |
|  | | No |
|  | | Yes |
